# The contribution of motor vehicle emissions to ambient fine particulate matter public health impacts in New York City: a health burden assessment

**DOI:** 10.1186/s12940-016-0172-6

**Published:** 2016-08-26

**Authors:** Iyad Kheirbek, Jay Haney, Sharon Douglas, Kazuhiko Ito, Thomas Matte

**Affiliations:** 1 New York City Department of Health and Mental Hygiene, Bureau of Environmental Surveillance and Policy, 125 Worth Street, Third Flr. CN-34E, New York, NY 10013 USA; 2 ICF International, 101 Lucas Valley Road, Suite 260, San Rafael, CA 94903 USA

**Keywords:** Fine particulate matter (PM2.5), Community multiscale air quality model (CMAQ), BenMAP, Traffic, Health impact assessment, Air quality management

## Abstract

**Background:**

On-road vehicles are an important source of fine particulate matter (PM_2.5_) in cities, but spatially varying traffic emissions and vulnerable populations make it difficult to assess impacts to inform policy and the public.

**Methods:**

We estimated PM_2.5_-attributable mortality and morbidity from on-road vehicle generated air pollution in the New York City (NYC) region using high-spatial-resolution emissions estimates, air quality modeling, and local health incidence data to evaluate variations in impacts by vehicle class, neighborhood, and area socioeconomic status. We developed multiple ‘zero-out’ emission scenarios focused on regional and local cars, trucks, and buses in the NYC region. We simulated PM_2.5_ concentrations using the Community Multi-scale Air Quality Model at a 1-km spatial resolution over NYC and combined modeled estimates with monitored data from 2010 to 2012. We applied health impact functions and local health data to quantify the PM_2.5_-attributable health burden on NYC residents within 42 city neighborhoods.

**Results:**

We estimate that all on-road mobile sources in the NYC region contribute to 320 (95 % Confidence Interval (CI): 220–420) deaths and 870 (95 % CI: 440–1280) hospitalizations and emergency department visits annually within NYC due to PM_2.5_ exposures, accounting for 5850 (95 % CI: 4020–7620) years of life lost. Trucks and buses within NYC accounted for the largest share of on-road mobile-attributable ambient PM_2.5_, contributing up to 14.9 % of annual average levels across 1-km grid cells, and were associated with 170 (95 % CI: 110–220) PM_2.5_-attributable deaths each year. These contributions were not evenly distributed, with high poverty neighborhoods experiencing a larger share of the exposure and health burden than low poverty neighborhoods.

**Conclusion:**

Reducing motor vehicle emissions, especially from trucks and buses, could produce significant health benefits and reduce disparities in impacts. Our high-spatial-resolution modeling approach could improve assessment of on-road vehicle health impacts in other cities.

**Electronic supplementary material:**

The online version of this article (doi:10.1186/s12940-016-0172-6) contains supplementary material, which is available to authorized users.

## Background

Fine particulate matter (PM_2.5_) is a common air pollutant that has been associated with multiple adverse health outcomes [1]. Despite declines in PM_2.5_ concentrations in New York City (NYC), recent estimates suggest ambient levels contribute to large numbers of avoidable premature deaths and diseases [2], and studies have shown a significant association between traffic-related air pollution and premature mortality [3]. Other studies have shown increased risk of respiratory and cardiovascular disease associated with close residential proximity to traffic pollution [4, 5].

Air quality public health impact analyses have emerged as an important approach for estimating the public health toll of air pollution, comparing its risks to other public health threats, and evaluating strategies and regulations designed to reduce exposures. Assessing source-specific contributions to health burdens can help prioritize strategies that offer the maximum benefit and minimize inequalities [6]. Typically, air quality and health modeling analyses performed for regulatory decision making or policy research are conducted at coarse spatial resolutions (e.g. 12-km, 36-km, county-level) [7–9]. However, analyses at these spatial scales do not allow researchers and policymakers to examine relationships between population health susceptibility and air pollution exposures, both of which can spatially vary substantively within a city at smaller scales. To address these limitations in the regulatory methodology, new modeling approaches are needed to combine information on within city disparities in both exposures and health.

While regulatory efforts have reduced emissions, on-road mobile sources continue to contribute to ambient levels of multiple air pollutants in NYC. Local source apportionment analyses conducted using data from the early 2000s suggested that 16–39 % of ambient PM_2.5_ concentrations in NYC were attributable to traffic sources [10, 11]. More recently, saturation sampling and land-use regression (LUR) modeling have demonstrated that traffic emissions density is an important contributor to within-city spatial variation in PM_2.5_, nitrogen dioxide (NO_2_) and black carbon levels in NYC [12]. While these studies provide useful information on the relative importance of local source sectors, source apportionment analyses using monitor data are limited by the locations of monitors, while LUR models that use surrogate indicators of emissions do not account for dispersion and chemical transformation processes and therefore may not be well-suited to quantify source contributions to ambient levels. Deterministic models of emissions, dispersion and chemical transformation processes can estimate exposure increments from individual sources, and recent developments in methods using high resolution modeling in urban areas can better represent spatial gradients across neighborhoods with wide variation in baseline health incidence [13, 14].

NYC, with high densities of populations living near emissions sources, also has the highest density of primary PM_2.5_ emissions among large US cities [15]. Wide variation in baseline health rates exist across the city, strongly associated with area-based poverty concentration [16]. Recent sustainability planning efforts in NYC have focused on reducing PM_2.5_ levels overall while shrinking disparities in exposures [17]. However, to date, limited data exist on the health burden from on-road mobile source emissions, the relative importance of regional as compared to local sources, or the differential contributions of differing vehicle classes. This information provides valuable context for developing and prioritizing local policy for cities.

To evaluate the extent and variation of PM_2.5_-attributable mortality and morbidity due to emissions of on-road mobile source primary PM_2.5_ and PM_2.5_ precursors in the region, we applied a local-scale air quality and health modeling framework to the five counties of NYC and the 28-county NYC metropolitan region. We estimated separately the PM_2.5-_attributable burden from emissions from all motor vehicle traffic in the region and within NYC, trucks and buses within NYC, cars within NYC, and on-road mobile sources in the region outside of NYC. We then explored the disparity in air quality and public health burden across neighborhoods of differing poverty status.

## Methods

**Fig. 1:**
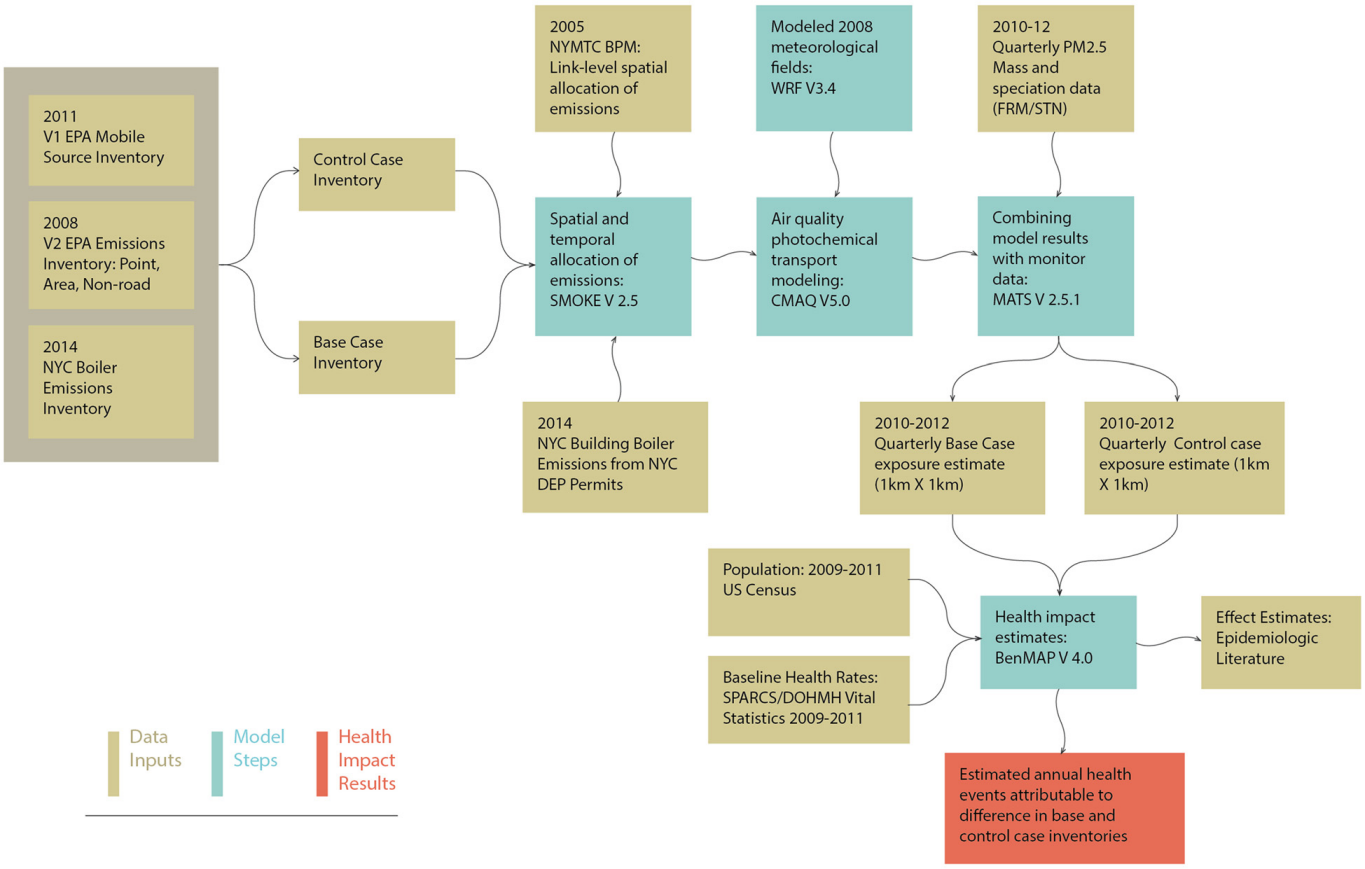
Data inputs and models for estimating the PM_2.5_-attributable public health burden from motor vehicles

We built an air quality and public health modeling framework for on-road mobile sources that included emissions inventory development and spatial allocation, meteorological and air quality modeling, combining modeling results and monitored data, and health impact calculations by modifying a prior framework used in an evaluation of heating fuel conversions in buildings [14, 18] (Fig. 1).

### Emissions inventory preparation

To characterize baseline conditions we built inventories from EPA’s 2008 National Emissions Inventory (NEI) modeling platform [19]. Described in detail elsewhere, we prepared emissions for three nested grids centered over NYC at 15-km national-scale, 5-km regional-scale, and 1-km local-scale horizontal resolution [18]. We replaced emissions for the on-road mobile source and building heating sectors in the 2008 NEI with more recent, refined local data to better reflect their spatial patterns.

We estimated on-road mobile source emissions using the most recently available county-level data from EPA’s 2011 National Emissions Inventory [20]. County-level emission estimates were spatially allocated to road links in proportion to modeled, link-level vehicle miles traveled from the 2005 New York State Metropolitan Transportation Council (NYMTC) Best Practices Model (BPM) [21]. Despite the relatively older time frame of the NYMTC BPM model, it provided the most recently available modeled counts for cars, trucks, and buses for links within the 28 counties in the NYC region and we assumed that relative spatial patterns in traffic density were reasonably stable between 2005 and 2011. To improve the spatial accuracy within the five NYC counties, the NYMTC shapefile was spatially aligned to the TeleAtlas street segment database within ArcGIS 9.2 Data & Maps.

For grid cells within the 1-km and 5-km modeling grids, we calculated on-road mobile source emissions of total volatile organic compounds (VOC), oxides of nitrogen (NO_x_), carbon monoxide (CO), sulfur dioxide (SO_2_), ammonia (NH_3_), primary PM_2.5_, and PM_2.5_ and VOC species profiles. Emissions were allocated do grid cells by first computing at each roadway link the vehicle miles traveled (VMT) by vehicle class (car, truck, bus) by multiplying the annual NYMTC vehicle-specific counts by the length of the segment. We then created ratios, by vehicle type, of the VMTs on each link to the total VMTs in the county. Second, we downloaded the on-road mobile source portion of the 2011 EPA NEI (V1) [20] and matched the Source Classification Code (SCC) subcategories to NYMTC car, truck, and bus categories. All light-duty and heavy-duty gasoline and diesel truck SCC codes were placed in the ‘truck’ category, while light-duty gasoline and diesel vehicles and motorcycles were included in the ‘car’ category. The heavy duty diesel bus SCC codes were included in the ‘bus’ category. Third, we estimated annual link-level emissions for each pollutant and vehicle type by multiplying the county-level emissions by the ratio of the VMTs on each link to the total VMTs in the county. Fourth, we created emissions totals for each pollutant/vehicle type in each 1-km and 5-km grid cell by summing the emissions from links that fell within each grid cell. For links that crossed multiple grid cells, emissions were apportioned based on the fraction of the link’s length included in each grid cell. Finally, because NYMTC does not include VMT breakdowns for categories within ‘car,’ ‘truck’, and ‘bus,’ we approximated these by extracting the county-level VMT data from EPA’s 2008 VMT database [22], then calculated the VMT fractions for gasoline, light- and heavy-duty diesel vehicles. This was then used to assign VOC and PM_2.5_ speciation profiles by estimating the VOC and PM_2.5_ emissions for gasoline, light- and heavy-duty diesel vehicles using the VMT fractions for each vehicle type, and then assigning the corresponding PM_2.5_ and VOC speciation profiles to the each of the categories.

To more accurately represent current building boiler emissions in NYC overall and the within the city, we updated the 2008 NEI for Nos. 2, 4, and 6 heating oil boilers using local permit data reflecting emissions as of 2015. These methods are described in detail elsewhere [14]. Briefly, emissions from Nos. 2, 4, and 6 boilers were calculated using EPA emissions factors [23] and NYC Department of Environmental Protection (NYCDEP) permit data that identify the location and heat throughput of the boiler. No. 4 emissions factors were adjusted to account for NYC-regulated 1500 ppm sulfur content, while No.2 emissions factors assumed a 15 ppm sulfur content, consistent with New York State-wide limits [24]. As many buildings are undergoing conversions of Nos. 4 and 6 boilers to comply with recent regulations [25], we reviewed the permit database as of December 2014, and assigned each building an annual emissions value based on the fuel they were using at that time and spatially allocated these emissions based on boiler address. To estimate emissions from No.2 boilers below the permitting threshold (350,000 Btu), we used NEI emissions not accounted for in the permits, allocating to buildings using surrogate data on building area and the county-specific percent of buildings using No.2 heating oil.

We prepared CMAQ-ready emissions by merging estimates for biogenic sources and all anthropogenic sectors with the updated on-road mobile source inventory and fuel oil boiler inventories. These emissions were processed using the Sparse Matrix Operator Kernel Emissions processor software (version 3.1) to create the air quality modeling input for the base case. We created three additional inventories reflecting removal of specific source categories: zeroing out all motor vehicle emissions within NYC (Sc1), zeroing out truck and bus emissions within NYC (Sc2), and zeroing out all motor vehicles in the 23 counties that surround the five NYC counties (Sc3).

### Air quality modeling

Detailed discussion of the application and evaluation of the meteorological and air quality modeling system has been presented elsewhere [18]. In short, meteorological fields for all grids were developed for 2008 using the Weather Research and Forecasting Model (WRF). Air quality modeling was conducted using the Community Multi-Scale Air Quality Model (CMAQ) version 5.0. Annual CMAQ simulations were conducted separately for the base case and each of the three zero-out scenarios and we utilized the daily simulated PM_2.5_ mass and species concentrations from the 1-km grid cells over NYC for subsequent health burden analyses.

### Health burden analysis

Exposure estimates at a 1-km resolution were developed using EPA’s Modeled Attainment Test Software (MATS) [26]. MATS combines the CMAQ modeled output with monitored PM_2.5_ mass and speciation data to create combined spatial surfaces, providing exposure estimates that use the monitor data but leverages the CMAQ simulated values to better estimate spatial gradients and surface response to changes in emissions. We developed 3 year, quarterly average estimates based on 2010–2012 EPA federal reference monitors (FRM) and speciation trends network (STN) monitors and the daily CMAQ modeling.

We computed the change in number of health events due to changes in PM_2.5_ concentrations between the base case and each of the three scenarios (Sc1, Sc2, and Sc3) using health impact functions [27, 28]. We applied risk functions for all-cause mortality from chronic exposure among those above 30 years of age [29], emergency department visits for asthma from acute exposure among all age groups (seasonally-specific risk estimates) [30], hospitalizations for all respiratory outcomes from acute exposure among those above 20 years of age (seasonally specific risk estimates for populations above 65 years of age) [31, 32], and hospitalizations for all cardiovascular outcomes from acute exposure among those above 40 years of age (seasonally specific risk estimates) [33]. Risk functions were chosen based on those determined to be most relevant to current New York City populations by selecting those published in peer-reviewed scientific journals and favoring those conducted in New York City when possible. We utilized NYC-specific risk functions for PM_2.5_-attributable emergency department visits for asthma and hospitalizations for cardiovascular disease. When local studies were not available, we used recent large, multi-city studies or those included in EPA risk analyses [34]. Baseline health data were obtained for 2009–2011 from the NYC Department of Health and Mental Hygiene Bureau of Vital Statistics and the New York Statewide Planning and Research Cooperative System, summarized across 22 age and sex groups within each of 42 United Hospital Fund (UHF) neighborhoods (zip code aggregates). Additional details on risk estimate selection and baseline health data is described elsewhere [27]. Population data for the same age/sex/neighborhood groups were calculated based on the US Census Bureau Population Estimate program [35]. We estimated 3 year, quarterly average health impacts of each of the scenarios within each of the 42 neighborhoods and summed the quarterly estimates to produce annual burdens. All health impact calculations were performed on a quarterly basis using EPA’s Benefits Mapping and Analysis Program (BenMAP) version 4.067 [36]. Further detail on our methodological choices for estimating the public health burden of PM_2.5_ on NYC residents can be found elsewhere [27].

To estimate years of life expectancy lost (YLL) we calculated life expectancy for 5 year age groupings using the city-wide, baseline mortality rates and standard abridged life table methods from the Centers for Disease Control and Prevention [37]. Years of life lost due to exposures associated with each scenario were calculated by multiplying the number of deaths in each age group attributable to the change in PM_2.5_ by the remaining life expectancy, then summing across all ages.

We first report the impacts on a citywide basis of removing all traffic in the 28-county region (adding Sc1 heath impacts to Sc3 health impacts), all traffic within NYC (Sc1), trucks and buses within NYC (Sc2), cars within NYC (subtracting Sc2 health impacts from Sc1 health impacts) and traffic from sources within the region but outside of NYC (Sc3). We explore correlations between on-road mobile source category contributions to ambient PM_2.5_ and neighborhood poverty then grouped neighborhoods based on percent of population residing under the federal poverty threshold (Low: 0–10 %, Medium: 10–20 %, High: 20–30 %, and Very High >30 %), calculated using the 2008–2012 American Community Survey. We report gradients in PM_2.5_ concentrations, rates of PM_2.5_-attributable health outcomes, and percent contribution to the total number of health events by neighborhood poverty level.

## Results

Emissions from motor vehicles within NYC produced 1817 tons of primary PM_2.5_, 43,934 tons of NO_x_, 20,613 tons of total VOCs, and 336 tons of SO_2_, annually, accounting for 17.5, 38.3, 21.9, and 4.6 % of all local pollutant emissions, respectively. Of the primary PM_2.5_ emissions produced by motor vehicles, the majority are produced by trucks and buses, accounting for 12.8 % of all local primary PM_2.5_ emissions. Based on the CMAQ model alone, primary PM_2.5_ concentrations attributable to truck and bus emissions within NYC contributed to an average of 27 % of total PM_2.5_ concentrations from all on-road mobile sources in the region. Secondarily generated PM_2.5_ from truck and bus precursor emissions within NYC accounted for an average of 12 % of total PM_2.5_ concentrations from all on-road mobile sources in the region (Additional file 1: Table S1). Cars within NYC contributed to an average of 10 and 25 % of total PM_2.5_ concentrations from all on-road mobile sources in the region due to primary and secondarily formed PM_2.5_, respectively (Additional file 1: Table S1). Based on link level NYMTC estimates within NYC, cars contributed 94 % of city VMTs while trucks and buses accounted for 6 %.

**Fig. 2:**
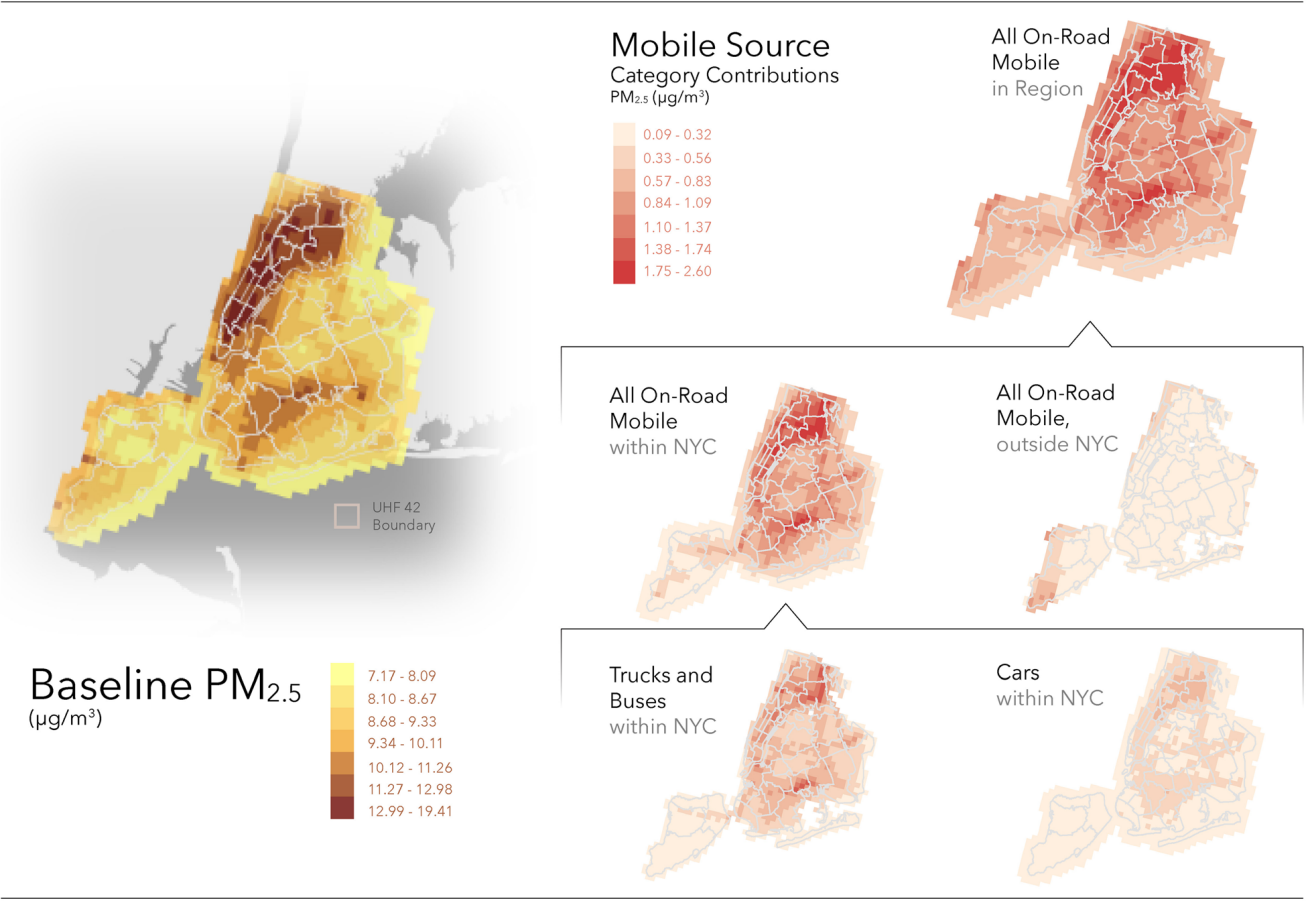
Estimated PM_2.5_ levels in the Base Case and contributions to ambient levels from on-road mobile source categories (1-km resolution)

Based on the combined model and monitor exposure surface, we estimate that traffic in the 28-county area contributed 0.38 to 2.60 μg/m^3^ across 1-km grid cells within NYC, accounting for 3.9 to 22.7 % of ambient PM_2.5_ levels (Fig. 2). Trucks and buses within NYC showed the largest within city contributions to ambient levels, accounting for 0.0 to 1.71 μg/m^3^ of PM_2.5_, or 0.0 to 14.9 % of PM_2.5_ concentrations. Emissions from cars within NYC and regional traffic (outside NYC) showed less of a contribution, with regional traffic mainly impacting grid cells along the edges of the city.

We estimate that, each year, emissions from on-road mobile sources within the five NYC counties contribute to 260 (95 % CI: 180, 340) PM_2.5_-attributable deaths from chronic PM_2.5_ exposure and 720 (95 % CI: 380, 1050) PM_2.5_-attributable emergency room visits and hospital admissions due to respiratory and cardiovascular outcomes from acute exposure (Table 1). Among these, emissions from buses and trucks account for the largest share of the city-wide burden, contributing to 170 (95 % CI: 110, 220) PM_2.5_-attributable deaths each year while cars contributed to 100 (95 % CI: 70, 120) PM_2.5_-attributable deaths each year. On-road mobile sources in the metropolitan area outside of the five NYC counties contribute to an additional 60 (95 % CI: 40, 80) PM_2.5_-attributable deaths each year and 150 (95 % CI: 70, 220) PM_2.5_-attributable morbidity outcomes each year. Overall, we estimate PM_2.5_ exposures from on-road mobile sources in the metropolitan region contribute to 320 (95 % CI: 220, 420) PM_2.5_-attributable deaths each year within NYC, contributing to 5850 (95 % CI: 4020, 7680) life years lost annually. The confidence intervals reported here only reflect those from the risk estimates derived from the epidemiologic studies and do not account for uncertainties in the other analysis steps.

**Table 1:** City-wide PM_2.5_-attributable health burdens of on-road mobile source emissions

	Count (95 % CI), percent of all events (95 % CI), percent of PM_2.5_-attributable events (95 % CI)				
	All motor vehicles in metropolitan region (Sc1 health impacts plus Sc3 health impacts)	All motor vehicles in NYC (Sc1 health impacts)	Buses and trucks in NYC (Sc2 health impacts)	Cars in NYC (Sc1 health impacts minus Sc2 health impacts)	All motor vehicles outside NYC (Sc3 impacts)
Emergency Room Visits, Respiratory (All Ages, acute exposure)	660 (380, 940), 0.76 % (0.44 %, 1.1 %), 13.11 % (7.5 %, 18.6 %)	550 (320, 780), 0.64 % (0.37 %, 0.90 %), 10.94 % (6.3 %, 15.5 %)	360 (210, 510), 0.42 % (0.24 %, 0.59 %), 7.19 % (4.17 %, 10.12 %)	190 (100, 270), 0.22 % (0.12 %, 0.31 %), 3.75 % (1.98 %, 5.36 %)	110 (60, 160), 0.13 % (0.07 %, 0.19 %), 2.17 % (1.19 %, 3.17 %)
Hospital Admissions, Cardiovascular (Ages 40 and above, acute exposure)	90 (20, 150), 0.14 % (0.03 %, 0.25 %), 13.32 % (3.1 %, 23.0 %)	70 (20, 120), 0.12 % (0.03 %, 0.20 %, 10.94 % (3.1 %, 18.4 %)	40 (10, 80), 0.07 % (0.02 %, 0.13 %), 6.84 % (1.53 %, 12.27 %)	30 (10, 50), 0.04 % (0.02 %, 0.08 %), 4.09 % (1.53 %, 7.67 %)	20 (4, 30), 0.03 % (0.01 %, 0.05 %), 2.41 % (0.61 %, 4.60 %)
Hospital Admissions, Respiratory (Ages 20 and above, acute exposure)	120 (40, 190), 0.27 % (0.09 %, 0.45 %), 12.96 % (4.5 %, 21.3 %)	100 (40, 150), 0.22 % (0.09 %, 0.35 %), 10.68 % (4.48 %, 16.8 %)	60 (20, 100), 0.14 % (0.05 %, 0.24 %, 6.80 % (2.24 %, 11.21 %)	30 (10, 60), 0.08 % (0.02 %, 0.14 %), 3.88 % (1.12 %, 6.73 %)	20 (10, 30), 0.05 % (0.02 %, 0.07 %), 2.28 % (1.12 %, 3.36 %)
Premature Mortality (Ages 30 and above, chronic exposure)	320 (220, 420), 0.68 % (0.47 %, 0.89 %), 13.22 % (9.14 %, 17.44 %)	260 (180, 340), 0.55 % (0.38 %, 0.72 %), 10.81 % (7.48 %, 14.12 %)	170 (110, 220), 0.35 % (0.23 %, 0.47 %), 6.86 % (4.57 %, 9.14 %)	100 (70, 120), 0.2 % (0.15 %, 0.26 %), 3.95 % (2.91 %, 4.98 %)	60 (40, 80), 0.12 % (0.09 %, 0.17 %), 2.41 % (1.67 %, 3.32 %)
Years of Life Lost (Ages 30 and above, chronic exposure)	5850 (4020, 7680)	4800 (3300, 6300)	3050 (2090, 4000)	1750 (1210, 2300)	1050 (720, 1380)

**Fig. 3:**
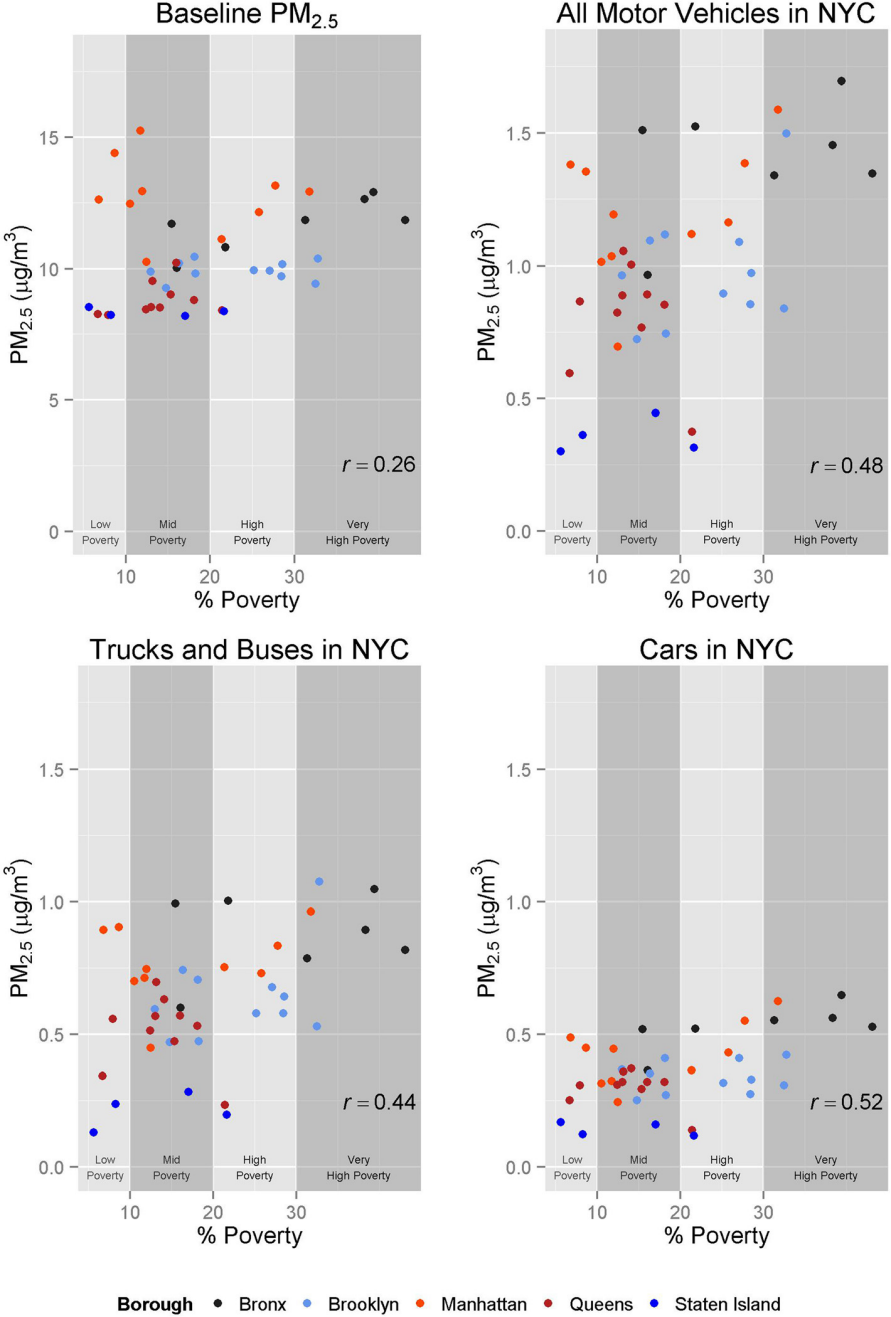
Correlations of estimated baseline PM_2.5_ concentrations and contributions from on-road mobile sources with neighborhood poverty metrics

We observed only a weak relationship between baseline PM_2.5_ concentrations and neighborhood poverty status, due to variable levels of PM_2.5_ across high income neighborhoods (Fig. 3). Affluent neighborhoods in NYC include many densely developed areas in Manhattan with high source density as well as more suburban neighborhoods with fewer emissions in Staten Island and Queens. However, there is a stronger relationship between on-road mobile-source-attributable PM_2.5_ and neighborhood poverty. This relationship is consistent for both bus/truck-attributable PM_2.5_ and car-attributable PM_2.5_, although a steeper gradient is found for bus/truck-attributable PM_2.5_ (average absolute difference in impact between low and high poverty neighborhoods of 0.36 μg/m^3^ for trucks/buses and 0.22 μg/m^3^ for cars), reflecting high densities of truck traffic in low-income neighborhoods.

There are large disparities in PM_2.5_-attributable health outcomes across neighborhoods with variable poverty status (Table 2). Across all source categories, higher mobile source PM_2.5_-attributable rates of morbidity and mortality are found in high poverty neighborhoods as compared to low poverty neighborhoods. This is due to the large disparity in the underlying rates of morbidity and mortality and higher on-road mobile source impacts on PM_2.5_ concentrations. The widest disparities are found for PM_2.5_-attributable emergency department visits for asthma. On-road mobile sources in the region contribute to rates of PM_2.5_-attributable asthma emergency department visits that are 8.3 times higher in the very high poverty neighborhoods relative to low poverty neighborhoods, due to high source density and relatively high asthma morbidity rates in these communities. The percent of incidences due to on-road mobile sources, which reflect the impacts of sources on neighborhood PM_2.5_ levels, also showed disparities across neighborhoods of varying poverty status, with higher percentages in lower income neighborhoods across all source categories, except regional traffic emissions outside of NYC. Regional traffic emissions did not produce large gradients in the percent of incidences across neighborhoods of varying poverty status due to relatively even impacts on PM_2.5_ concentrations across the city, with slightly higher impacts on PM_2.5_ concentrations in some grid cells along the edges of the City in higher income neighborhoods of Manhattan and the northern Bronx.

**Table 2:** Distribution of PM_2.5_-attributable health outcomes due to on-road mobile sources by area poverty

Metric	Source sector	Low poverty	Medium poverty	High poverty	Very high poverty
		(*n* = 6)	(*n* = 19)	(*n* = 10)	(*n* = 7)
Impacts on PM_2.5_ Concentrations (μg/m^3^, percent of ambient concentrations)	All on-road mobile sources in region	1.09 (10.9 %)	1.14 (11.2 %)	1.21 (11.6 %)	1.64 (14.0 %)
	All on-road mobile sources in NYC	0.81 (8.1 %)	0.94 (9.2 %)	0.97 (9.3 %)	1.39 (11.9 %)
	Trucks and buses in NYC	0.51 (5.1 %)	0.60 (5.9 %)	0.62 (6.0 %)	0.87 (7.5 %)
	Cars in NYC	0.30 (3.0 %)	0.33 (3.3 %)	0.35 (3.3 %)	0.52 (4.5 %)
	All on-road mobile sources outside NYC	0.28 (2.8 %)	0.21 (2.0 %)	0.24 (2.3 %)	0.25 (2.1 %)
Impacts on Mortality among residents above 30 years of age PM_2.5_-attributable rate per 100,000 residents (95 % CI), Percent of all events (95 % CI), Percent of PM_2.5_-attributable events (95 % CI)	All on-road mobile sources in region	5.27 (3.62, 6.92) 0.58 % (0.40 %, 0.76 %) 11.8 % (8.1 %, 15.4 %)	5.86 (4.03, 7.69) 0.63 % (0.43 %, 0.82 %) 12.6 % (8.7 %, 16.6 %)	7.36 (5.06, 9.66) 0.7 % (0.48 %, 0.92 %) 13.7 % (9.4 %, 17.9 %)	8.98 (6.17, 11.78) 0.88 % (0.60 %, 1.15 %) 15.3 % (10.5 %, 20.1 %)
	All on-road mobile sources in NYC	3.92 (2.69, 5.14) 0.43 % (0.30 %, 0.57 %) 8.7 % (6 %, 11.5 %)	4.81 (3.31, 6.32) 0.52 % (0.35 %, 0.68 %) 10.4 % (7.1 %, 13.6 %)	6.03 (4.14, 7.92) 0.57 % (0.39 %, 0.75 %) 11.2 % (7.7 %, 14.7 %)	7.6 (5.22, 9.97) 0.74 % (0.51 %, 0.97 %) 12.9 % (8.9 %, 17 %)
	Trucks and buses in NYC	2.51 (1.72, 3.29) 0.28 % (0.19 %, 0.36 %) 5.6 % (3.8 %, 7.3 %)	3.07 (2.11, 4.03) 0.33 % (0.23 %, 0.43 %) 6.6 % (4.5 %, 8.7 %)	3.85 (2.65, 5.06) 0.37 % (0.25 %, 0.48 %) 7.2 % (4.9 %, 9.4 %)	4.73 (3.25, 6.20) 0.46 % (0.32 %, 0.61 %) 8 % (5.5 %, 10.6 %)
	Cars in NYC	1.41 (0.97, 1.85) 0.16 % (0.11 %, 0.20 %) 3.1 % (2.2 %, 4.1 %)	1.75 (1.20, 2.29) 0.19 % (0.13 %, 0.25 %) 3.8 % (2.6 %, 4.9 %)	2.18 (1.50, 2.86) 0.21 % (0.14 %, 0.27 %) 4 % (2.8 %, 5.3 %)	2.87 (1.98, 3.77) 0.28 % (0.19 %, 0.37 %) 4.9 % (3.4 %, 6.4 %)
	All on-road mobile sources outside NYC	1.35 (0.93, 1.78) 0.15 % (0.10 %, 0.20 %) 3.0 % (2.1 %, 4.0 %)	1.05 (0.72, 1.37) 0.11 % (0.08 %, 0.15 %) 2.3 % (1.5 %, 3.0 %)	1.33 (0.91, 1.74) 0.13 % (0.09 %, 0.17 %) 2.5 % (1.7 %, 3.2 %)	1.38 (0.95, 1.81) 0.13 % (0.09 %, 0.18 %) 2.4 % (1.6 %, 3.1 %)
Impacts on Emergency Department Visits for Asthma among all residents (PM_2.5_-attributable rate per 100,000 residents (95 % CI), Percent of all events (95 % CI) Percent of PM_2.5_-attributable events (95 % CI)	All on-road mobile sources in region	2.39 (1.4, 3.39) 0.64 % (0.37 %, 0.9 %) 10.8 % (6.3 %, 15.3 %)	4.64 (2.71, 6.58) 0.7 % (0.41 %, 1 %) 12 % (7 %, 17 %)	9.54 (5.51, 13.56) 0.76 % (0.44 %, 1.09 %) 13.2 % (7.6 %, 18.7 %)	19.97 (11.37, 28.56) 0.83 % (0.47 %, 1.18 %) 14.2 % (8.1 %, 20.3 %)
	All on-road mobile sources in NYC	1.79 (1.04, 2.53) 0.48 % (0.28 %, 0.67 %) 8.1 % (4.7 %, 11.4 %)	3.87 (2.26, 5.47) 0.59 % (0.34 %, 0.83 %) 10 % (5.8 %, 14.1 %)	7.89 (4.57, 11.19) 0.63 % (0.37 %, 0.9 %) 10.9 % (6.3 %, 15.5 %)	16.96 (9.66, 24.23) 0.7 % (0.4 %, 1 %) 12.1 % (6.9 %, 17.3 %)
	Trucks and buses in NYC	1.17 (0.7, 1.65) 0.31 % (0.19 %, 0.44 %) 5.3 % (3.2 %, 7.5 %)	2.58 (1.54, 3.62) 0.39 % (0.23 %, 0.55 %) 6.7 % (4 %, 9.4 %)	5.21 (3.09, 7.32) 0.42 % (0.25 %, 0.59 %) 7.2 % (4.3 %, 10.1 %)	10.98 (6.42, 15.54) 0.45 % (0.27 %, 0.64 %) 7.8 % (4.6 %, 11.1 %)
	Cars in NYC	0.61 (0.34, 0.88) 0.16 % (0.09 %, 0.23 %) 2.8 % (1.5 %, 4 %)	1.29 (0.72, 1.85) 0.19 % (0.11 %, 0.28 %) 3.3 % (1.8 %, 4.8 %)	2.68 (1.48, 3.87) 0.21 % (0.12 %, 0.31 %) 3.7 % (2 %, 5.3 %)	5.97 (3.25, 8.69) 0.25 % (0.13 %, 0.36 %) 4.3 % (2.3 %, 6.2 %)
	All on-road mobile sources outside NYC	0.61 (0.36, 0.86) 0.16 % (0.1 %, 0.23 %) 2.7 % (1.6 %, 3.9 %)	0.78 (0.45, 1.1) 0.12 % (0.07 %, 0.17 %) 2 % (1.2 %, 2.8 %)	1.65 (0.94, 2.36) 0.13 % (0.08 %, 0.19 %) 2.3 % (1.3 %, 3.3 %)	3.02 (1.7, 4.33) 0.12 % (0.07 %, 0.18 %) 2.1 % (1.2 %, 3.1 %)
Impacts on Hospitalizations for Cardiovascular Disease among residents over 40 years of age. (PM_2.5_-attributable rate per 100,000 residents (95 % CI), Percent of all events (95 % CI) Percent of PM_2.5_-attributable events (95 % CI)	All on-road mobile sources in region	1.59 (0.39, 2.78) 0.12 % (0.03 %, 0.21 %) 11.5 % (2.8 %, 20.3 %)	2.12 (0.52, 3.71) 0.13 % (0.03 %, 0.23 %) 12.7 % (3.1 %, 22.3 %)	2.74 (0.68, 4.8) 0.15 % (0.04 %, 0.26 %) 13.7 % (3.4 %, 24 %)	3.81 (0.96, 6.66) 0.18 % (0.05 %, 0.32 %) 15.3 % (3.8 %, 26.7 %)
	All on-road mobile sources in NYC	1.15 (0.28, 2.02) 0.09 % (0.02 %, 0.15 %) 8.4 % (2.1 %, 14.7 %)	1.75 (0.43, 3.07) 0.11 % (0.03 %, 0.19 %) 10.5 % (2.6 %, 18.4 %)	2.25 (0.56, 3.94) 0.12 % (0.03 %, 0.22 %) 11.2 % (2.8 %, 19.7 %)	3.23 (0.81, 5.64) 0.15 % (0.04 %, 0.27 %) 12.9 % (3.2 %, 22.6 %)
	Trucks and buses in NYC	0.72 (0.17, 1.28) 0.05 % (0.01 %, 0.09 %) 5.3 % (1.2 %, 9.3 %)	1.1 (0.26, 1.94) 0.07 % (0.02 %, 0.12 %) 6.6 % (1.6 %, 11.6 %)	1.42 (0.34, 2.5) 0.08 % (0.02 %, 0.14 %) 7.1 % (1.7 %, 12.5 %)	1.99 (0.48, 3.49) 0.1 % (0.02 %, 0.17 %) 8 % (1.9 %, 14 %)
	Cars in NYC	0.43 (0.11, 0.75) 0.03 % (0.01 %, 0.06 %) 3.1 % (0.8 %, 5.4 %)	0.65 (0.17, 1.13) 0.04 % (0.01 %, 0.07 %) 3.9 % (1 %, 6.8 %)	0.83 (0.22, 1.45) 0.05 % (0.01 %, 0.08 %) 4.2 % (1.1 %, 7.2 %)	1.24 (0.33, 2.15) 0.06 % (0.02 %, 0.1 %) 5 % (1.3 %, 8.6 %)
	All on-road mobile sources outside NYC	0.43 (0.1, 0.76) 0.03 % (0.01 %, 0.06 %) 3.2 % (0.8 %, 5.5 %)	0.37 (0.09, 0.65) 0.02 % (0.01 %, 0.04 %) 2.2 % (0.6 %, 3.9 %)	0.49 (0.12, 0.86) 0.03 % (0.01 %, 0.05 %) 2.5 % (0.6 %, 4.3 %)	0.58 (0.15, 1.01) 0.03 % (0.01 %, 0.05 %) 2.3 % (0.6 %, 4.1 %)
Impacts on Hospitalizations for Respiratory Disease among residents above 20 years of age (PM_2.5_-attributable rate per 100,000 residents(95 % CI), Percent of all events (95 % CI) Percent of PM_2.5_-attributable events (95 % CI)	All on-road mobile sources in region	1.13 (0.42, 1.85) 0.55 (0.22, 0.89) 11 % (4.1 %, 18 %)	1.44 (0.55, 2.34) 0.25 % (0.09 %, 0.4 %) 12.1 % (4.6 %, 19.6 %)	2.04 (0.8, 3.29) 0.28 % (0.11 %, 0.46 %) 13.4 % (5.2 %, 21.5 %)	3.58 (1.43, 5.71) 0.33 % (0.13 %, 0.52 %) 14.7 % (5.9 %, 23.5 %)
	All on-road mobile sources in NYC	0.84 (0.31, 1.37) 0.16 % (0.06 %, 0.27 %) 8.1 % (3 %, 13.3 %)	1.19 (0.45, 1.93) 0.2 % (0.08 %, 0.33 %) 10 % (3.8 %, 16.1 %)	1.69 (0.66, 2.71) 0.23 % (0.09 %, 0.38 %) 11 % (4.3 %, 17.7 %)	3.02 (1.21, 4.83) 0.28 % (0.11 %, 0.44 %) 12.4 % (5 %, 19.8 %)
	Trucks and buses in NYC	0.54 (0.2, 0.88) 0.11 % (0.04 %, 0.17 %) 5.3 % (1.9 %, 8.6 %)	0.77 (0.29, 1.24) 0.13 % (0.05 %, 0.21 %) 6.4 % (2.4 %, 10.4 %)	1.08 (0.42, 1.74) 0.15 % (0.06 %, 0.24 %) 7.1 % (2.8 %, 11.4 %)	1.87 (0.75, 2.99) 0.17 % (0.07 %, 0.27 %) 7.7 % (3.1 %, 12.3 %)
	Cars in NYC	0.3 (0.11, 0.48) 0.06 % (0.02 %, 0.1 %) 2.9 % (1.1 %, 4.7 %)	0.42 (0.16, 0.69) 0.07 % (0.03 %, 0.12 %) 3.5 % (1.3 %, 5.8 %)	0.6 (0.23, 0.97) 0.08 % (0.03 %, 0.13 %) 3.9 % (1.5 %, 6.3 %)	1.15 (0.46, 1.84) 0.11 % (0.04 %, 0.17 %) 4.7 % (1.9 %, 7.5 %)
	All on-road mobile sources outside NYC	0.3 (0.11, 0.48) 0.06 % (0.02 %, 0.09 %) 2.9 % (1.1 %, 4.7 %)	0.25 (0.09, 0.41) 0.04 % (0.02 %, 0.07 %) 2.1 % (0.8 %, 3.4 %)	0.36 (0.14, 0.58) 0.05 % (0.02 %, 0.08 %) 2.4 % (0.9 %, 3.8 %)	0.55 (0.22, 0.89) 0.05 % (0.02 %, 0.08 %) 2.3 % (0.9 %, 3.6 %)
Baseline Outcome Rates (rate per 100,000 residents)	All-cause mortality (ages 30 and above)	907.5	934.2	1051.1	1024.6
	Emergency department visits for asthma (all ages)	374.9	659.5	1248.4	2416.1
	Hospitalizations for cardiovascular disease (ages 40 and above)	1354.9	1589.3	1824.5	2089.1
	Hospitalizations for respiratory disease (ages 20 and above)	508.9	589.0	722.8	1089.0

## Discussion

In this study, we applied a high-spatial-resolution modeling framework to assess the impacts of on-road mobile source generated primary PM_2.5_ and PM_2.5_ precursors on NYC populations. We estimated that over 300 deaths each year in the five counties of NYC are due to PM_2.5_ exposures related to motor vehicle emissions in the 28-county region, contributing to 5850 YLL annually. These exposures also contribute to almost 900 emergency department visits and hospitalizations for respiratory and cardiovascular disease annually. Overall, on-road mobile sources contribute to 0.7 % of all deaths in NYC each year and 13.2 % of PM_2.5_-attributable deaths, with the largest share of this impact due to emissions from trucks and buses on NYC roadways. Within NYC, we observed wide variation in incremental ambient PM_2.5_ contributions from traffic across 1-km grid cells. The largest impacts on air quality levels and health outcomes are found in the highest poverty areas of the city due to overlapping patterns of traffic density (particularly truck traffic) and higher underlying baseline incidence of morbidity.

Comparative analysis of traffic types demonstrated that trucks and buses, despite a much lower share of overall VMT within the city, contribute to the largest share of the on-road mobile source air quality burden with the majority of the primary PM_2.5_ emissions coming from heavy duty diesel trucks and buses. We found that these sources contribute to 7.5 % of the ambient levels of PM_2.5_ in high poverty neighborhoods and up to 0.6 % of all deaths in the most affected neighborhood. Traffic from counties in the region outside of NYC showed less of an impact on local PM_2.5_ concentrations, which were evenly distributed across neighborhoods of varying poverty status.

Prior work in other cities and nationally has also explored the air quality and public health impacts of traffic. A nationwide analysis indicted that all mobile sources (including all non-road, aircraft, locomotive, marine vessels, and ocean-going vessels) could contribute to 17,000 PM_2.5_-attributable deaths in 2016 [6]. Other research has pointed to the importance of traffic-related PM_2.5_ on mortality, suggesting that in 2005 traffic emissions contributed to 3000 PM_2.5_-attributable deaths nationally [8]. Applying a similar air quality and health modeling framework as was used in this analysis, additional research has suggested significant regional benefits to eliminating motor vehicle trips [38]. Prior natural experiments on removal of traffic in urban areas during events have shown some associated improvements in air quality, although the benefits are often pollutant specific and vary based on the situation being studied, particularly when evaluating pollutants with strong regional contributions such as PM_2.5_ [39, 40]. Source apportionment analyses conducted using data from the early 2000s from a few monitoring locations in the region suggested that 16–39 % of ambient PM_2.5_ concentrations in NYC are attributable to traffic sources [10, 11]. These estimates are higher than those found in this analysis, potentially due to the limitations in the numbers of monitors used in the source-apportionment studies (where monitoring sites are skewed to high emissions locations) and newer traffic emissions estimates that reflect lower emissions from on-road mobile sources in more recent years. To our knowledge this is the first analysis in this region that explicitly examines impacts of differing types of vehicles at a high spatial resolution across neighborhoods within an urban area, which provides valuable insight when exploring effective emissions control strategies.

This analysis also provides a new perspective on variation in PM_2.5_ exposures across populations of differing socioeconomic status (SES). Prior work has found that higher SES communities in NYC experience higher overall PM_2.5_ and NO_2_ exposures, due to the confluence of building and traffic sources in high-income areas, a pattern that is unusual among major metropolitan areas where lower SES areas often experience higher pollutant exposures [41, 42]. In contrast to the pattern for total PM_2.5_, on-road mobile source-attributable PM_2.5_ concentrations are higher in low-income neighborhoods of the city, indicating that efforts to reduce exposures in these burdened communities should be focused on on-road mobile source-related programs.

We find that measures to reduce emissions from heavy-duty vehicles within NYC should be prioritized, particularly those traveling roadways in neighborhoods with high densities of susceptible populations and low income residents. Studies conducted in other cities have shown success implementing congestion charging schemes or low-emissions zones that target the most polluting trucks and buses [43, 44], with differing observations on the distribution of benefits by socioeconomic status, depending on the location evaluated [45, 46]. In designing congestion mitigation schemes, this analysis suggests a focus on NYC as a whole and on heavy-duty diesel vehicles would yield significantly greater health benefits, as opposed to focusing on vehicles in the most congested urban core. Measures to reduce VMTs and emissions from trucks and buses within the city may need to address trips from all types of vehicles originating inside and outside of the city. For example, in the Hunt’s Point section of the South Bronx, an area with high burdens of PM_2.5_-attributable morbidity and mortality from truck emissions, an estimated 57 % of trucks servicing the meat and produce market (one of the largest food distribution centers in the world) came from outside of the city [47]. Other surveys have suggested 20 % of car miles traveled in NYC are from trips originating outside of the city [48]. While direct emissions from cars have less of an impact on air quality and health compared to heavy duty diesel vehicles, car trips contribute to congestion, which increases diesel emissions on routes shared with trucks and buses.

While this study offers new insights and methods for assessing PM_2.5_-attributable health impacts, there are some limitations. The confidence intervals described in our results reflect only the confidence intervals reported in the risk estimates derived from the epidemiologic studies and do not account for uncertainties in the other steps of the analysis. EPA’s inventory estimates are subject to uncertainties in emissions factors, vehicle mix, and activity. Despite likely simulating spatial gradients in emissions better than other commonly used surrogates such as road density, they may not fully account for higher emissions in low speed stop-and-go traffic within the congested urban core. Future work would benefit for more precise estimates of emissions at a neighborhood-level. The meteorological and air quality simulations also carry uncertainties common in these types of studies. Prior evaluation of the base case modeling, however, showed that the WRF and CMAQ models performed within recommended bias and precision benchmarks [18]. A strength of our study is that it employs 1-km PM_2.5_ exposure modeling, a higher resolution than prior studies of this type and thus better accounts for within-city variations in susceptibility. This provides new methods and insight into how source-specific impacts can vary within an urban area and among populations of differing socioeconomic status. Despite this, 1-km resolution may not fully capture microscale, near-roadway exposures that can vary within several hundred meters of the roadway [49, 50].

Our health impact estimates include common limitations described elsewhere [28] some of which have been addressed by using neighborhood-level health outcome data. We have utilized epidemiological studies that assume uniform relative risk across all neighborhoods with varying traffic density. Emerging research has suggested stronger associations between asthma morbidity and air pollutant exposures in higher traffic areas (implying that PM_2.5_ emissions from traffic may be more toxic), and such effect modification research is a field of ongoing study [51]. Similarly, while our analyses applied risk estimates based on total PM_2.5_ exposures, recent analyses of the ACS cohort has suggested higher chronic mortality risk associated with PM_2.5_ with higher sulfur content [52]. As more evidence accumulates we will evaluate the sensitivity of our burden estimates to varying risk functions, and future work will evaluate how variations in neighborhood-level risk contribute to disparities in impacts across the City. While this analysis has leveraged associations between PM_2.5_ and excess emergency department visits and hospitalizations, studies have shown that air pollution exposures can also contribute to new cases of asthma [53], suggesting morbidity estimates in this analysis are conservative. Finally, our analysis focused only on the impacts of on-road mobile source emissions on PM_2.5_ associated mortality and select cardio-respiratory outcomes and does not account for the wide range of additional negative effects of motor vehicle traffic and congestion, including health effects associated with other pollutants and noise, contributions to greenhouse gas emissions, risk of pedestrian and other injury, and time wasted.

## Conclusion

Local scale air quality and public health modeling can provide valuable information on the contribution of sources to pollution-attributable health and disparity within an urban area. In this study, we presented a methodology for assessing the public health impacts of traffic in cities, and evaluating these impacts across populations with varying underlying health and socioeconomic status. In applying these methods in NYC, we found that air pollutant emissions from on-road mobile sources contribute to hundreds of preventable PM_2.5_-attributable deaths, hospitalizations, and emergency department visits among residents of NYC, with disproportionate impacts in high poverty neighborhoods, indicating that increased policy efforts should focus on the most polluting vehicles in these neighborhoods.

## Additional file

Additional file 1: Table S1. Contributions of source categories to primary and secondary PM_2.5_ levels at grid cells within New York City. (DOCX 13 kb)

## Abbreviations

BenMAP: Environmental Benefits Mapping and Analysis Program
BPM: Best Practices Model
CI: Confidence interval
CMAQ: Community Multi-scale Air Quality Model
CO: Carbon monoxide
DEP: New York City Department of Environmental Protection
EPA: US Environmental Protection Agency
FRM: Federal reference monitors
LUR: Land use regression
NEI: National Emissions Inventory
NH_3_: Ammonia
NO_2_: Nitrogen dioxide
NO_x_: Oxides of nitrogen
NYC: New York City
NYMTC: New York State Metropolitan Transportation Council
PM_2.5_: Fine particulate matter
SCC: Source classification code
SES: Socioeconomic status
SO_2_: sulfur dioxide
STN: Speciation trends network
UHF: United Hospital Fund
VMT: vehicle miles traveled
VOC: Volatile organic compounds
WRF: Weather research forecasting model
YLL: Years of life expectancy lost
