# Supplementary material for: The contribution of motor vehicle emissions to ambient fine particulate matter public health impacts in New York City: a health burden assessment
Source: Environ Health. 2016 Aug 26;15(1):89. doi: 10.1186/s12940-016-0172-6 (PMC5002106; doi:10.1186/s12940-016-0172-6)
Supplement: Additional file 1: Table S1. — Contributions of source categories to primary and secondary PM2.5 levels at grid cells within New York City. (DOCX 13 kb) [file 12940_2016_172_MOESM1_ESM.docx]

Supplemental Table 1

Table S1: Contributions of source categories to primary and secondary PM_2.5_ levels at grid cells within New York City^i^

|  |  |  | **Total PM_2.5_**  μg/m^3^ concentration average, (range of 1km grid cells) | **Primary PM_2.5_**  μg/m^3^ concentration average, (range of 1km grid cells)  *percent of total PM_2.5_ concentrations from all mobile sources in the region, average (range across 1-km gridcells)* | **Secondary PM_2.5_**  μg/m^3^ concentration average, (range of 1km grid cells  *percent of total PM_2.5_ concentrations from all mobile sources in the region, average (range across 1-km gridcells)* |
| --- | --- | --- | --- | --- | --- |
| **All Mobile Sources in the Region** | | | 2.23 (0.94, 4.62) | 1.12 (0.44, 2.63) | 1.11 (0.48, 2.1) |
|  | **All Mobile Sources in NYC** | | 1.71 (0.21, 4.11) | 0.87 (0.12, 2.45)  *37% (6%, 57%)* | 0.84 (0.08, 1.83)  *36% (4%, 45%)* |
|  |  | *cars* | 0.8 (0.09, 1.87) | 0.23 (0.03, 0.59)  *10% (2%, 15%)* | 0.57 (0.06, 1.33)  *25% (3%, 34%)* |
|  |  | *trucks/buses* | 0.91 (0.12, 2.71) | 0.64 (0.09, 2.06)  *27% (4%, 48%)* | 0.27 (0.03, 0.69)  *12% (1%, 18%)* |
|  | **All Mobile Sources Outside NYC** | | 0.52 (0.36, 1.86) | 0.25 (0.17, 1.28)  *13% (4%, 62%)* | 0.27 (0.19, 0.7)  *14% (5%, 38%)* |

^i^Based on simulations from the CMAQ model alone
